# Evaluation of the Effect of MTAD on Expression of Enterococcus faecalis Virulence Factors Considering the Role of Different Obturating Materials

**Published:** 2018-11

**Authors:** Behnam Bolhari, Abbas Bahador, Mehrfam Khoshkhounejad, Mahsa Sobhi Afshar, Mohammad Moghaddaszadeh

**Affiliations:** 1 Associate Professor, Department of Endodontics, School of Dentistry, Tehran University of Medical Sciences, Tehran, Iran; Laser Research Center in Dentistry, Tehran University of Medical Sciences, Tehran, Iran; 2 Associate Professor, Department of Microbiology, School of Medicine, Tehran University of Medical Sciences, Tehran, Iran; 3 Assistant Professor, Department of Endodontics, School of Dentistry, Tehran University of Medical Sciences, Tehran, Iran; 4 Private Practice, Tehran, Iran

**Keywords:** Enterococcus faecalis, Gutta-Percha, MTAD, Resilon Sealer, Virulence Factors

## Abstract

**Objectives:**

The aim of this study was to determine the effect of MTAD on the expression of virulence factors of Enterococcus faecalis (E.faecalis) considering the role of Guttapercha/AH26 or Resilon/RealSeal SE as root canal obturating materials.

**Materials and Methods:**

One-hundred and forty-four single-rooted human teeth were instrumented to a standardized apical size. Root canals were infected by E.faecalis (ATCC 29212). Ninety teeth were irrigated with MTAD and randomly divided into three groups. In two groups, root canals were obturated by either Gutta-percha/AH26 or Resilon/RealSeal SE. Root canals were kept unobturated in the third group. The remaining 54 teeth received no final irrigation. All groups were then subdivided into three timepoint subgroups in which dentin powder was obtained from each sample to determine the expression of specific virulence factors of E.faecalis (*efa, esp, gel, fsr*) using real-time reverse transcription polymerase chain reaction (RT-PCR). Statistical analysis was performed by one-way analysis of variance (ANOVA) and Tukey’s post-hoc test. The statistical power was set at P-value ≤0.05.

**Results:**

MTAD was effective against the expression of most of the tested virulence factors, and Gutta-percha/AH26 increased the antibacterial efficacy of MTAD.

**Conclusions:**

MTAD could inhibit the expression of some known virulence factors of E.faecalis at the majority of tested timepoints. This may partly explain some of the mechanisms of antimicrobial efficacy of MTAD against this resistant microorganism which is known as one of the main causes of failure of root canal treatment.

## INTRODUCTION

Successful root canal therapy depends on eliminating bacteria from the root canal system and preventing reinfection by establishing a high-quality obturation and maintaining a tight bacterial coronal seal [1]. The persistent presence of bacteria in seemingly well-obturated root canals can impair the healing process after root canal treatment. An effective antimicrobial irrigant, preferably with a long-acting effect, as well as a proper filling material can lead to more successful results in eliminating bacterial contamination [1]; however, some filling materials may affect the antimicrobial efficacy of root canal irrigating solutions [2].

Enterococcus faecalis (E.faecalis) is one of the most resistant microorganisms against antimicrobial irrigants and intracanal medicaments, which can endure nutritional deprivation situations and has been predominantly associated with persistent periapical infections in necrotic pulps and endodontically-treated teeth [3]. It has been speculated that the clinical resistance of this microorganism is partly related to some of its virulence factors [4].

These virulence factors may play a significant role in the adherence of E.faecalis to host tissues, its invasiveness, and abscess formation, as well as secretion of different products that enhance biofilm formation. They also can affect host inflammatory responses [5]. These virulence factors include aggregation substance (***AS***), serine protease (***sprE***), enterococcal polysaccharide antigen (***epa***), gelatinase (***gelE***), enterococcal surface protein (***esp***), and general stress protein (***gls24***); however, ***gelE***, ***sprE***, and ***esp*** have not been all systematically found in clinical isolates [6].

E.faecalis endocarditis antigen (***efaA***) is a very potent virulence factor which can be found in E.faecalis strains detected in root canals of treatment-resistant endodontic infections [7], and it was first identified from the antiserum of a patient with E.faecalis endocarditis [8]. The amino acid sequence of the associated protein ***efaA*** showed a major homology to a group of streptococci proteins known as adhesins [8], and it has been proven that expression of endocarditis virulence factor ***efaA*** can be induced by the growth of E.faecalis in a medium containing serum [9].

E.faecalis ***esp*** has been known as a contributing factor in the colonization and persistence of infection. It is suggested that ***esp*** promotes biofilm formation; however, additional factors may possibly contribute to biofilm formation in E.faecalis [10].

The gelatinase (***gelE***) of E.faecalis is an extracellular zinc metalloprotease that can hydrolyze collagen, gelatin, and casein [11]. Gelatinase and serine protease (***sprE***) are encoded in an operon, ***gelE***-***sprE***, which its expression is positively regulated by a quorum-sensing system encoded by the ***fsr*** locus [12]. It has been suggested that gelatinase enhances biofilm formation and can promote the aggregation of bacterial cells in microcolonies to form a primary attachment site with subsequent development into a three-dimensional (3D) structure [13]. Also, its enzymatic activity is required for its role in biofilm formation [13].

The aforementioned ***fsr*** quorum-sensing system controls biofilm development through the production of gelatinase [13]. It has been suggested that E.faecalis ***fsr*** can affect biofilm formation independently from gelatinase, and its effect is similar to that of ***agr*** in Staphylococci [14]. Many studies have focused on evaluating the antimicrobial efficacy of different irrigating solutions against E.faecalis. MTAD, a relatively new irrigant, consisting of doxycycline, citric acid, and tween 80 (as a detergent), has been claimed to be effective in killing E.faecalis even when diluted [15]. Some researchers have focused on the mechanisms of the antimicrobial efficacy of MTAD and the probable interactions between this root canal irrigant and the obturation material of choice [2]. Bonding of doxycycline to dentin may be affected by some root filling materials and reduce the antimicrobial efficacy of MTAD over time [16,17].

Gutta-percha, in combination with different sealers, is the most commonly used root canal filling material [18]. Resilon is a synthetic polymer-based thermoplastic root canal filling material [19].

It was first introduced in 2004 as a biocompatible material with a superior sealing ability through bonding to dentin. Although there are few research studies on clinical applications of this material, it has been found that its obturation quality and clinical outcome are similar to those of gutta-percha [19].

The aim of this study was to evaluate the effect of MTAD on the expression of the virulence factors of E.faecalis in human root canal dentin, using real-time reverse transcription polymerase chain reaction (RT-PCR), before and after obturation with Gutta-percha/AH26 or Resilon/RealSeal SE.

## MATERIALS AND METHODS

One-hundred and forty-four non-carious, single-rooted, single- canalled human anterior maxillary teeth with relatively straight roots were selected. All the teeth used in this study have been extracted due to periodontal problems and were not maintainable. The participants were informed that their teeth might be used for experimental processes, and informed consent was obtained orally. This research has been approved by the Ethics Committee on Research of Tehran University of Medical Sciences (IR.TUMS.REC.1395.2356).

The anatomy of the roots was confirmed by taking straight and angulated radiographic images, and teeth with anatomical abnormalities and calcified canals were excluded. After immersion in 1.3% sodium hypochlorite (NaOCl) for 20 minutes, the root surfaces were debrided using a periodontal curette and then access cavities were prepared. Afterwards, working lengths were determined using a #15 K-file (Dentsply Maillefer, Tulsa, OK, USA), and root canals were instrumented up to apical size #35 by Mtwo rotary instruments (VDW, Munich, Germany) according to the manufacturer’s instruction. Canal irrigation was performed with 2 ml of sterile saline between each file. To prevent bacterial leakage, the apical foramina were then sealed with sticky wax, and the root surfaces were covered with two layers of nail varnish. Specimens were sterilized by gamma radiation (40 kiloGrays (kGy); ISO standard 11137).

Then, a suspension of E.faecalis (ATCC 29212) with an optical density (OD) of 0.3–0.4 (Novaspec II, Pharmacia Biotech, Sweden) at 450-nm wavelength was obtained from an overnight culture of E.faecalis (10^6^ CFU (colony-forming units)/ml) in brain heart infusion (BHI) broth. The suspension was injected into the root canals, and the samples were incubated at 37°C and 100% humidity for one week. After that, 90 teeth were finally irrigated with MTAD according to the manufacturer’s instruction (BioPure® MTAD® Root Canal Cleanser, Dentsply International Inc., Tulsa, OK, USA). The teeth were initially rinsed with 3 ml of 1.3% NaOCl for 20 minutes. Then, 1 ml of MTAD (Dentsply Tulsa, Tulsa, OK, USA) was left in the root canals for 5 minutes and slightly agitated using a #15 K-file. Then, a final rinse by 4 ml of MTAD was provided, and the canals were dried using sterile paper points (AriaDent, Tehran, Iran) which were sterilized by gamma radiation (40 kGy; ISO standard 11137). The teeth were then randomly divided into three groups (n=30). In group G/M, the canals were obturated by gutta-percha (Gapadent Co. Ltd., Incheon, Korea) and AH26 sealer (DeTrey, Dentsply, Konstanz, Germany) using the lateral condensation technique.

In group R/M, obturation was performed by Resilon/RealSeal SE (Pentron Clinical Technologies, Wallingford, CT, USA) using the same obturation technique, and the samples were light-cured for 40 seconds (LED.D Guilin Woodpecker Medical Instrument Co. Ltd., China). Root canals in group M were kept unobturated as positive controls. The remaining 54 teeth received no final irrigation; from these, 30 teeth were not filled (group D as negative controls), and the remaining 24 teeth were divided into two subgroups (n=12) according to the obturating material and were filled either with Gutta-percha/AH26 (group G) or Resilon/RealSeal SE (group R) using the aforementioned protocol. Then, the access cavities of all teeth were sealed using Coltosol (AriaDent, Tehran, Iran). Teeth in all groups were then subdivided into 1-, 3-, and 6-week timepoint subgroups.

During the research period, all specimens were incubated at 37°C and 100% humidity.

After the mentioned time intervals, teeth in each subgroup were sectioned parallel to their longitudinal axis using a diamond disc (DiaDent, Maribor, Slovenia) and a spatula under aseptic conditions, and the root filling material was gently removed. Dentin powder was obtained from the middle thirds of the root canals using a low-speed handpiece and a #5 round bur (Teeskavan, Tehran, Iran).

The obtained dentin powder was then evaluated to determine the expression of specific virulence factors of E.faecalis by the real-time RT-PCR method.

### RNA extraction:

Extraction of RNA was performed using the mRNA-ONLY ™ prokaryotic mRNA isolation kit (Epicenter; Illumina Inc., San Diego, CA, USA) according to the manufacturer’s instructions. The obtained RNA was refrigerated at a temperature of −70°C for the synthesis of cDNA.

### cDNA production:

The cDNA synthesis was performed according to the RevertAid first-strand cDNA synthesis kit protocols (Fermentas, Vilnius, Lithuania). In order to control the cDNA, its expression was investigated by the RT-PCR method and 16S rRNA primers.

### Real-Time RT-PCR:

The real-time RT-PCR technique was used to study the expression of the target genes. The real-time RT-PCR reaction was performed using 23S rRNA and 16S rRNA primers (housekeeping genes) as normalizers, genes No. 1, 2, 3, and 4 (Table 1), and the real-time RT-PCR ready-reaction mixture (Master Mix; Takara Bio USA Inc., CA, USA). Triplicate reactions were done on each sample. Using the data from the real-time RT-PCR and the ddCt formula, the ratio of changes in the expression of target genes was calculated in comparison with the control sample. Statistical analysis was performed by one-way analysis of variance (ANOVA) and Tukey’s post-hoc test. The confidence level was set at P-value ≤0.05.

**Table 1.** Primer sequences of the genes related to Enterococcus faecalis (E.faecalis) virulence factors

	** Gene **	** Forward primer **	** Reverse primer **	** Tm (°C) **	** Amplicon size (bp) **
** 1 **	fsrC	GCTTATTTGGAAGAACAACGTAT	CGAAACATCGCTAGCTCT	58.70	100
		CAA	TCGT	61.04	
** 2 **	gelE	CGGAACATACTGCCGGTTTAGA	TGGATTAGATGCACCCGA	60.42	100
			AAT	57.42	
** 3 **	esp	GGAACGCCTTGGTATGCTAAC	GCCACTTTATCAGCCTGA	59.33	94
			ACC	59.25	
** 4 **	efa	TGGGACAGACCCTCACGAATA	CGCCTGTTTCTAAGTTCA	60.27	100
			AGCC	60.10	
** 5 **	23S rRNA	CCTATCGGCCTCGGCTTAG	AGCGAAAGACAGGTGAG	59.41	100
			AATCC	60.35	
** 6 **	16S rRNA	CCGAGTGCTTGCACTCAATTGG	CTCTTATGCCATGCGGCA	62.65	137
			TAAAC	60.61	

Tm=Melting Temperature, bp=Base Pair

## RESULTS

The expression of E.faecalis virulence factors at 1-, 3-, and 6-week intervals was investigated. The studied virulence factors included ***efa***, ***esp***, ***gel***, and ***fsr***. The time of checking each virulence factor is given as a number after its name; for example, ***efa1*** denotes the expression of ***efa*** virulence factor after one week.

Table 2 shows the mean values related to the expression of E.faecalis virulence factors and reveals the effect of MTAD through the comparison of the mean values between the study groups at 1-, 3-, and 6-week timepoints. P-values are demonstrated in Table 3.

**Table 2.** Descriptive values (means and standard deviations) of expression of tested virulence factors in all of the studied subgroups.

** VF groups **		** efa1 **	** efa3 **	** efa6 **	** esp1 **	** esp3 **	** esp6 **	** gel1 **	** gel3 **	** gel6 **	** fsr1 **	** fsr3 **	** fsr6 **
D	mean±SD	4.33±.055	3.82±.189	4.46±.091	9.65±.105	10.29±.151	9.09±.140	2.81±.196	2.60±.070	2.79±.085	1.34±.043	1.17±.140	1.25±.135
M		2.97±.097	2.97±.168	3.87±.065	5.25±.115	8.14±.680	8.14±.130	2.01±.110	2.35±.105	2.58±.121	0.89±.212	0.93±.102	1.10±.130
G/M	mean±SD	2.02±.172	2.61±.294	3.47±.098	2.39±.180	6.95±.127	7.69±.180	1.23±.196	1.97±.156	2.47±.105	0.49±.211	0.81±.132	0.94±.104
R/M		2.89±.240	3.06±.050	3.77±.165	5.15±.125	7.69±.385	8.03±.090	1.98±.102	2.15±.124	2.58±.087	0.88±.246	0.92±.092	1.05±.135
M	mean±SD	2.97±.097	2.97±.168	3.87±.065	5.25±.115	8.14±.680	8.14±.130	2.01±.110	2.35±.105	2.58±.121	0.89±.212	0.93±.102	1.10±.130
G/M		2.02±.172	2.61±.294	3.47±.098	2.39±.180	6.95±.127	7.69±.180	1.23±.196	1.97±.156	2.47±.105	0.49±.211	0.81±.132	0.94±.104
M	mean±SD	2.97±.097	2.97±.168	3.87±.065	5.25±.115	8.14±.680	8.14±.130	2.01±.110	2.35±.105	2.58±.121	0.89±.212	0.93±.102	1.10±.130
R/M		2.89±.240	3.06±.050	3.77±.165	5.15±.125	7.69±.385	8.03±.090	1.98±.102	2.15±.124	2.58±.087	0.88±.246	0.92±.092	1.05±.135
R	mean ±SD	4.26±.095	3.79±.121	4.46±.160	9.44±.081	10.10±.151	8.89±.081	2.78±.061	2.62±.213	2.76±.133	1.29±.040	1.15±.052	1.24±.117
D		4.33±.055	3.82±.189	4.46±.091	9.65±.105	10.29±.151	9.09±.140	2.81±.196	2.60±.070	2.79±.085	1.34±.043	1.17±.140	1.25±.135
G	mean ±SD	3.96±.149	3.51±.086	4.53±.088	8.97±.076	9.74±.195	8.94±.208	2.60±.128	2.53±.045	2.79±.020	1.19±.108	1.01±.182	1.21±.126
D		4.33±.055	3.82±.189	4.46±.091	9.65.105	10.29±.151	9.09±.140	2.81±.196	2.60.070	2.79±.085	1.34±.043	1.17±.140	1.25±.135
R	mean±SD	4.26±.095	3.79±.121	4.46±.160	9.44±.081	10.10±.151	8.89±.081	2.78±.061	2.62±.213	2.76±.133	1.29±.040	1.15±.052	1.24±.117
G		3.96.±149	3.51±.086	4.53±.088	8.97±.076	9.74±.195	8.94±.208	2.60±.128	2.53±.045	2.79±.020	1.19±.108	1.01±.182	1.21±.126
G	mean±SD	3.96±.149	3.51.086	4.53±.088	8.97±.076	9.74±.195	8.94±.208	2.60±.128	2.53±.045	2.79±.020	1.19±.108	1.01±.182	1.21±.126
G/M		2.02±.172	2.61±.294	3.47±.098	2.39±.180	6.95±.127	7.69±.180	1.23±.196	1.97±.156	2.47±.105	0.49±.211	0.81±.132	0.94±.104
R	mean±SD	4.26±.095	3.79±.121	4.46±.160	9.44±.081	10.10±.151	8.89±.081	2.78±.061	2.62±.213	2.76±.133	1.29±.040	1.15±.052	1.24±.117
R/M		2.89±.240	3.06±.050	3.77±.165	5.15±.125	7.69±.385	8.03±.090	1.98±.102	2.15±.124	2.58±.087	0.88±.246	0.92±.092	1.05±.135
M	mean±SD	2.97±.097	2.97±.168	3.87±.065	5.25±.115	8.14±.680	8.14±.130	2.01±.110	2.35±.105	2.58±.121	0.89±.212	0.93±.102	1.10±.130
G		3.96±.149	3.51±.086	4.53±.088	8.97±.076	9.74±.195	8.94±.208	2.60±.128	2.53±.045	2.79±.020	1.19±.108	1.01±.182	1.21±.126
M	mean±SD	2.97±.097	2.97±.168	3.87±.065	5.25±.115	8.14±.680	8.14±.130	2.01±.110	2.35±.105	2.58±.121	0.89±.212	0.93±.102	1.10±.130
R		4.26±.095	3.79±.121	4.46±.160	9.44±.081	10.10±.151	8.89±.081	2.78±.061	2.62±.213	2.76±.133	1.29±.040	1.15±.052	1.24±.117

**Table 3.** P values of comparing the expression of tested virulence factors in the studied subgroups.

	** VF **	** efa1 **	** efa3 **	** efa6 **	** esp1 **	** esp3 **	** esp6 **	** gel1 **	** gel3 **	** gel6 **	** fsr1 **	** fsr3 **	** fsr6 **

** Groups **													
** Comparison groups **	** D **	0.00 ^ ** ^	0.00 ^ ** ^	0.00 ^ ** ^	0.00 ^ ** ^	0.00 ^ ** ^	0.00 ^ ** ^	0.00 ^ ** ^	0.24	0.16	0.05	0.25	0.69
	** M **												

** Comparison groups **	** G/M **	0.00 ^ ** ^	0.06	0.07	0.00 ^ ** ^	0.15	0.11	0.00 ^ ** ^	0.58	0.74	0.11	0.87	0.88
	** R/M **												

** Comparison groups **	** M **	0.00 ^ ** ^	0.17	0.01 ^ * ^	0.00 ^ ** ^	0.01 ^ * ^	0.02 ^ * ^	0.00 ^ ** ^	0.03 ^ * ^	0.79	0.09	0.82	0.63
	** G/M **												

** Comparison groups **	** M **	0.98	0.98	0.88	0.89	0.60	0.91	1.00	0.46	1.00	1.00	1.00	0.99
	** R/M **												

** Comparison groups **	** R **	0.99	1.00	1.00	0.32	0.98	0.59	1.00	1.00	0.99	0.99	1.00	1.00
	** D **												

** Comparison groups **	** G **	0.07	0.30	0.97	0.00 ^ ** ^	0.41	0.81	0.50	0.98	1.00	0.87	0.60	0.99
	** D **												

** Comparison groups **	** R **	0.19	0.41	0.97	0.00 ^ * ^	0.79	0.99	0.63	0.96	0.99	0.97	0.73	1.00
	** G **												

** Comparison groups **	** G **	0.00 ^ ** ^	0.00 ^ ** ^	0.00 ^ ** ^	0.00 ^ ** ^	0.00 ^ ** ^	0.00 ^ ** ^	0.00 ^ ** ^	0.00 ^ * ^	0.02 ^ * ^	0.00 ^ * ^	0.42	0.16
	** G/M **												

** Comparison groups **	** R **	0.00 ^ ** ^	0.00 ^ * ^	0.00 ^ ** ^	0.00 ^ ** ^	0.00 ^ ** ^	0.00 ^ ** ^	0.00 ^ ** ^	0.00 ^ * ^	0.31	0.08	0.28	0.50
	** R/M **												

** Comparison groups **	** M **	0.00 ^ ** ^	0.02 ^ * ^	0.00 ^ ** ^	0.00 ^ ** ^	0.00 ^ ** ^	0.00 ^ ** ^	0.00 ^ * ^	0.55	0.16	0.31	0.97	0.89
	** G **												

** Comparison groups **	** M **	0.00 ^ ** ^	0.00 ^ ** ^	0.00 ^ ** ^	0.00 ^ ** ^	0.00 ^ ** ^	0.00 ^ ** ^	0.00 ^ ** ^	0.19	0.27	0.10	0.34	0.77
	** R **												

A significant increase in the expression of ***efa*** virulence factor in group D in comparison with group M indicated that MTAD could effectively decrease the expression of ***efa*** at the three time intervals of 1, 3, and 6 weeks (Tables 2 and 3).

The expression of ***efa*** factor was greater in R/M group in comparison with G/M group at the three timepoints, but the difference was significant only at the 1-week timepoint (Tables 2 and 3).

Comparing the expression of ***efa*** factor between groups M and G/M showed that Guttapercha/AH26 have boosted the antimicrobial effect of MTAD at the mentioned time intervals, which was statistically significant at 1-week and 6-week timepoints.

There was no significant difference in the expression of ***efa*** virulence factor between R/M and M groups at the tested time intervals, indicating that Resilon/RealSeal SE could not improve the negative effect of MTAD on the expression of ***efa*** virulence factor (Tables 2 and 3).

The significantly greater expression of ***esp*** virulence factor in group D compared to group M at the tested timepoints indicated the effectiveness of MTAD in reducing the expression of this virulence factor (Tables 2 and 3).

Although the expression of ***esp*** virulence factor was greater in R/M samples than in G/M samples, the difference was significant only at the 1-week time interval (Tables 2 and 3).

Moreover, the significantly higher expression of ***esp*** factor in group M than in group G/M indicated that Gutta-percha/AH26 might have increased the inhibitory effect of MTAD on the expression of this virulence factor at all timepoints (Tables 2 and 3).

The expression of ***esp*** factor was not significantly different between groups R/M and M at any of the time intervals, indicating that Resilon/RealSeal SE had not affected the impact of MTAD on the expression of this virulence factor (Tables 2 and 3).

The expression of ***gel*** factor in group D was greater than that in group M at all the timepoints but only showed a statistically significant difference at the 1-week timepoint. This may be indicative of a diminution in the inhibitory effect of MTAD on the expression of this virulence factor over time (Tables 2 and 3).

Although the expression of ***gel*** was higher in group R/M than in group G/M, the difference was not statistically significant, except at the 1-week timepoint (Tables 2 and 3).

The expression of ***gel*** factor was greater in group M than in group G/M at all the tested timepoints, but showed a statistically significant difference only at 1-week and 3-week intervals, which suggested that Gutta-percha/AH26 could have enhanced the inhibitory effect of MTAD on the expression of this virulence factor, which would supposedly reduce over time (Tables 2 and 3).

A nonsignificant difference in the expression of ***gel*** virulence factor between groups R/M and M indicated that Resilon/RealSeal SE had not impacted the effect of MTAD on the expression of this virulence factor at any of the time intervals (Tables 2 and 3).

Comparing the expression of ***fsr*** factor between D and M groups indicated a nonsignificant difference at all the tested timepoints. This finding could suggest the ineffectiveness of MTAD on the expression of this virulence factor during the experiment period (Tables 2 and 3).

No significant difference was found between G/M and R/M groups at any of the time intervals regarding the expression of ***fsr*** factor (Tables 2 and 3). There was also no significant difference in the expression of this virulence factor at any of the mentioned timepoints between groups G/M and M as well as groups R/M and M (Tables 2 and 3).

These findings indicated that neither Guttapercha/AH26 nor Resilon/RealSeal SE might influence the effect of MTAD on the expression of ***fsr*** factor (Tables 2 and 3).

Furthermore, the comparison of group D with groups R and G showed no significant changes in the expression of the virulence factors at the majority of the timepoints.

The expressions of ***efa*** and ***esp*** factors were significantly higher at all the time intervals in group R in comparison with groups M and R/M. Also, there was a significantly higher expression of ***efa*** and ***esp*** factors at all the timepoints in group G in comparison with groups M and G/M.

## DISCUSSION

Among the Enterococcus species, E.faecalis is the most commonly isolated species from oral infections such as infected root canals [20], periradicular abscesses [21], and marginal periodontitis [22]. E.faecalis has been more commonly detected in cases of failed endodontic therapy rather than primary infections [23]. Most strains of E.faecalis have the ability to form a robust biofilm that its development may be modulated by the dominant environmental conditions [24].

Several potential virulence factors of E.faecalis have been recognized over the past few years. It has been speculated that these virulence factors could synergically increase bacterial virulence and cause a deeper bacterial invasion as well as greater tissue damage [25].

In this study, the antimicrobial efficacy of MTAD was evaluated in the presence and absence of two different obturation materials through determining the expression of certain virulence factors which have been speculated to play a role in the biofilm formation and pathogenicity of E.faecalis.

The efficacy of MTAD has been previously investigated by Torabinejad et al [15]; it has been proven that MTAD sustains its antimicrobial efficacy at a dilution of 1:200 and is the only irrigant which could eliminate E.faecalis in a period of 2 to 5 minutes in comparison with other disinfectant solutions such as NaOCl and ethylenediaminetetraacetic acid (EDTA) [15].

Newberry and colleagues [26] also showed that MTAD was able to inhibit the growth of 7 out of 8 strains of E.faecalis. Nevertheless, there are also some articles that show a low antibacterial efficacy for MTAD [27,28]. Some studies have shown that MTAD could significantly reduce the expression of E.faecalis virulence factors when used as an irrigating solution in root canal therapy. MTAD also has the ability to eliminate many other resistant microorganisms, and its superiority over other disinfectants has been shown in some studies [15,26].

In this study, the selected timepoints were based on the half-life of MTAD, which has been mentioned to be three weeks [29].

In the present study, irrigation of root canals with MTAD caused a significant reduction in the expression of ***efa***, ***esp***, and ***gel*** virulence factors in most of the tested time intervals when compared to non-irrigated root canals. Moreover, ***efa*** and ***esp*** factors were affected more than other virulence factors. ***fsr*** was the only virulence factor that its expression was not adversely affected by MTAD. This may indicate that MTAD might be unable to affect the mechanisms by which ***fsr*** can lead to biofilm formation in E.faecalis species. Although it is noteworthy that, based on the findings of this study, the expression of ***fsr*** was lower than other virulence factors in the tested strain of E.faecalis (ATCC 29212) among all the samples and the mentioned timepoints. This finding suggests that even MTAD, as a known effective irrigating solution, may provide a less profound antimicrobial effect in strains that predominantly express some specific virulence factors.

In this study, the expression of virulence factors was also evaluated after obturating the root canals with either Gutta-percha/AH26 or Resilon/RealSeal SE in order to assess the probable effect of these materials on the remaining antimicrobial effect of MTAD. It was shown that Gutta-percha/AH26 could significantly increase the effectiveness of MTAD according to the decline in the expression of ***efa***, ***esp***, and ***gel*** factors at most of the tested timepoints, while Resilon/RealSeal SE did not significantly affect the efficacy of MTAD.

Bolhari et al [1] have also previously shown that in the presence of Gutta-percha and AH26, the activity of MTAD was significantly greater than that in the presence of Resilon and RealSeal SE. The reason for this has been mentioned to be the effect of Resilon on reducing the pH of the surrounding environment and consequently declining the antimicrobial efficacy of MTAD [30]. It has also been mentioned that the increased efficacy of MTAD in the presence of gutta-percha could be attributed to the presence of zinc oxide in the composition of this obturation material, which was proven to have an antibacterial effect [31]. In order to define the solo effect of gutta-percha on the properties of MTAD, it is necessary to use it without any sealer. In the recent study, it was shown that there was less expression of specific virulence factors in the samples irrigated with MTAD and obturated with gutta-percha and AH26, which could suggest another mechanism for the boosting effect of Gutta-percha/AH26 as the obturating material of choice while irrigating root canals with MTAD.

Due to the time-dependent reduction of the tested virulence factors’ expression, it may be speculated that the antimicrobial efficacy of MTAD would diminish over time. Bolhari et al [32] reported similar results and showed that the substantivity of MTAD reduces over time regardless of whether or not the canal was filled. In the present study, in obturated canals, Guttapercha/AH26 and Resilon/RealSeal SE did not significantly affect the expression of E.faecalis virulence factors when the root canals were not irrigated by MTAD. Similar findings were reported by Shin et al [33] when Guttapercha/AH26 and Resilon/RealSeal SE did not completely inhibit the growth of E.faecalis. Therefore, it may be concluded that the reduction in the expression of E.faecalis virulence factors in this study can mainly be attributed to the antimicrobial effect of MTAD.

To the best of our knowledge, this is the first time that the antibacterial effect and substantivity of MTAD are evaluated by analyzing the expression of virulence factors of E.faecalis in human root canal dentin using real-time RT-PCR after obturation with Gutta-percha/AH26 or Resilon/RealSeal SE. Under the conditions of the current study, MTAD could inhibit the expression of some known virulence factors of E.faecalis at most of the tested timepoints, which may partly explain some of the mechanisms of the antimicrobial efficacy of this irrigant against this resistant microorganism which is known as one of the main causes of failure of root canal treatment. It is important to note that in the clinical situation, the root canal system is a polymicrobial environment containing a variety of different species which their interactive behavior may affect the antimicrobial efficacy of root canal irrigants. The need to create controlled conditions in ex-vivo studies means that researchers have to eliminate the possible interfering factors, and the results of this ex-vivo experimental study cannot be generalized to clinical conditions until further tested in clinical trials. There is no doubt that more clinical studies on the efficacy of this solution in comparison with other disinfecting irrigants are necessary.

## CONCLUSION

Considering the limitations of this study, it seems that the antimicrobial properties of MTAD and its substantivity can partly be attributed to its negative effect on the expression of some virulence factors of E.faecalis. In this regard, MTAD may be affected by different root canal filling materials. Therefore, if it is clinically proven, it may be advised to choose certain filling materials in conjunction with specific irrigation solutions in different clinical situations in order to achieve the best antimicrobial efficacy and a successful clinical outcome.
